# Fascin Is a Key Regulator of Breast Cancer Invasion That Acts via the Modification of Metastasis-Associated Molecules

**DOI:** 10.1371/journal.pone.0027339

**Published:** 2011-11-04

**Authors:** Monther Al-Alwan, Safiah Olabi, Hazem Ghebeh, Eman Barhoush, Asma Tulbah, Taher Al-Tweigeri, Dahish Ajarim, Chaker Adra

**Affiliations:** 1 Stem Cell Therapy Program, King Faisal Specialist Hospital and Research Centre, Riyadh, Saudi Arabia; 2 Department of Pathology and Laboratory Medicine, King Faisal Specialist Hospital and Research Centre, Riyadh, Saudi Arabia; 3 Department of Oncology, King Faisal Specialist Hospital and Research Centre, Riyadh, Saudi Arabia; 4 Transplantation Research Centre (TRC), Brigham and Women's Hospital and Children's Hospital Boston, Harvard Medical School, Boston, Massachusetts, United States of America; Institute of Molecular and Cell Biology, Singapore

## Abstract

The actin-bundling protein, fascin, is a member of the cytoskeletal protein family that has restricted expression in specialized normal cells. However, many studies have reported the induction of this protein in various transformed cells including breast cancer cells. While the role of fascin in the regulation of breast cancer cell migration has been previously shown, the underlying molecular mechanism remained poorly defined. We have used variety of immunological and functional assays to study whether fascin regulates breast cancer metastasis-associated molecules. In this report we found a direct relationship between fascin expression in breast cancer patients and; metastasis and shorter disease-free survival. Most importantly, in vitro interference with fascin expression by loss or gain of function demonstrates a central role for this protein in regulating the cell morphology, migration and invasion potential. Our results show that fascin regulation of invasion is mediated via modulating several metastasis-associated genes. We show for the first time that fascin down-regulates the expression and nuclear translocation of a key metastasis suppressor protein known as breast cancer metastasis suppressor-1 (BRMS1). In addition, fascin up-regulates NF-kappa B activity, which is essential for metastasis. Importantly, fascin up-regulates other proteins that are known to be critical for the execution of metastasis such as urokinase-type plasminogen activator (*uPA*) and the matrix metalloproteases (MMP)-2 and MMP-9. This study demonstrates that fascin expression in breast cancer cells establishes a gene expression profile consistent with metastatic tumors and offers a potential therapeutic intervention in metastatic breast cancer treatment through fascin targeting.

## Introduction

Breast cancer is one of the leading causes of cancer mortality in women worldwide. In spite of significant advances in cancer treatment, mortality results from local invasion and/or distant metastasis and not from tumor in the primary site [1], [2]. Therefore, there is a strong demand to understand the cellular and molecular mechanisms that regulate tumor invasion and metastasis in order to develop better treatment regimens. Metastasis is a multi-step process involving neovascularization, stromal invasion by cancer cells, and infiltration into vascular and lymphatic spaces, extravasation and growth at a secondary site [1]–[6]. Many cellular and molecular factors have been reported to regulate tumor metastasis (Reviewed in [7]). However, a critical step of metastatic tumor cells is the ability to cross extracellular matrix (ECM) tissue boundaries [8], [9], a process that can be accomplished by expression of active metalloproteases (MMPs) by cancer cells, which facilitate their migration. In addition, metastatic tumor cells can down-regulates metastatic suppressors such as the Breast Cancer Metastasis Suppressor 1 (BRMS1), which belongs to family of metastasis suppressors that suppress metastasis without blocking orthotopic tumor growth. BRMS1 has been reported to block lung and regional lymph node metastasis in experimental breast models [10] and decreased expression of this protein has been demonstrated to correlate with reduced disease-free survival in human breast cancer [11]. It was reported that BRMS1 suppression of tumor metastasis is mediated via inhibition of NF-kB and subsequent suppression of the urokinase-type plasminogen activator (*uPA*) [12], a serine protease that is known to activate the MMPs [13] leading to invasion and metastasis.

Manipulation of the actin cytoskeleton, leading to enhancement of cell motility, is one of the dominant cellular mechanisms that regulate metastasis [14]. Fascin is a member of the actin cytoskeletal proteins that bundles actin filaments into tertiary structures within dynamic cellular structures such as microspikes, stress fibers and membrane ruffles [15], consistent with its abundant expression at these sites. Interestingly, fascin expression is highly restricted to certain tissues such as brain and spleen and abundant fascin expression was reported in normal specialized cells such as neurons, glia cells, endothelial cells and antigen presenting dendritic cells [16]–[19].

It is widely believed that de-regulation of normal tissue organization and homeostasis including cell adhesion, motility and cytoskeleton can contribute to many human diseases including cancers [19]. In fact, fascin over-expression induces membrane protrusion and has been reported to enhance cell motility in various systems [20]. In addition to its expression in specialized normal cells, many studies have reported induction of fascin expression in various transformed cells. In many human carcinomas including breast cancer, fascin expression correlates with clinically aggressive tumors and metastasis [21]. However, the exact function of fascin and its regulation of other genes in breast cancer are still poorly understood.

Development of membrane protrusions by non-immune cells has been clearly demonstrated to enhance cell motility and to facilitate interactions with other cell types. Therefore, we proposed to investigate whether fascin expression in breast cancer cells is directly involved in promoting cell migration and invasion and to elucidate the molecular mechanism that regulate this process. Our results clearly demonstrated a key role for fascin in regulating both cell morphology and invasion. In addition, we have shown that fascin facilitates this process via modulation of metastasis-associated genes; specifically down-regulation of the metastasis suppressor BRMS1 and up-regulation of NF-κB activity as well as the induction of uPA, MMP-2, MMP-9 expression.

## Materials and Methods

### Cell lines

The human breast cancer cell line MDA-MB-231 (HTB-26) and T-47D (HTB-20) were purchased from ATCC and seeded in DMEM containing 10% FBS, 200 mM L-glutamine and antibiotic-antimycotic liquid (Invitrogen) at 37°C in a 5% CO2 humidified atmosphere.

### Antibodies and reagents

The mouse anti-human fascin mAb and APC-conjugated secondary Ab were from Dako and Jackson Laboratories, respectively. The mouse mAb to detect MMP-2 (2C1-1D12) and MMP-9 (2C3) were from Invitrogen and Chemicon, respectively. The mouse mAb (ab57082), rabbit polyclonal Ab (ab65244) to detect BRMS1, mAb to detect uPA (PGM2005) and mAB to detect TATA binding protein (TBP) were all from ABcam. Abs to detect p65 (C22B4), phopho-p65 (93H1), IκBα (L35A5) and phospho-IκBα (5A5), alpha-tubulin were all from Cell Signaling Technology. The GAPDH, β-actin, PCNA and p50 (C-19) were from Santa Cruz. The secondary anti-mouse HRP-conjugated and anti-goat HRP-conjugated were from Southern Biotech and Serotec, respectively. The Rhodamine-phalloidin to detect F-actin was from Invitrogen. The remaining antibodies were from BD Bioscience. Fascin specific-, negative control-SiRNA and GAP-DH-SiRNA were purchased from Applied Biosystem. Fascin specific-ShRNA and control were purchased from Thermo Scientific. The MMP-2/MMP-9 inhibitor II was from Calbiochem. The cell proliferation reagent WST-1 was purchased from Roche. The CSFE cell proliferation dye was purchased from Guava Biotechnologies, Inc.

### Flow cytometry and cell sorting

Cell permeablization and fascin staining were performed as previously described [22]. Fluorescence was analyzed using a flow cytometer (LSR II; BD, Mountain View, CA). Cell sorting was performed on GFP-Fascin transfected cells using cell sorter (FACS DIVA, BD, Mountain View, CA).

### Fascin knockdown

For transient knockdown, MDA-MB-231 cells were seeded in the presence of control SiRNA or Fascin (ID s13209) SiRNA as per instructions of Applied Biosystem. Maximum inhibition of fascin as assessed by western blot and FACS analysis was observed 72 hours post SiRNA treatment and thus this time was used for the rest of the study.

To rule out any off target effect of the used SiRNA, stable ShRNA clones were generated. For stable fascin knockdown, lentiviral vectors expressing fascin shRNA (clone Id: TRCN000012039) or control shRNA were obtained from Thermo Scientific. Recombinant lentivirus was produced in 293T cells as previous described [23] and mixed with MDA-MB-231 cells in the presence of polybrene (10 µg/ml) for 16 hour at 37°C, medium was replaced and cultured for additional 72 hours. Transduced cells were selected with puromycin followed by cloning and screening for the lack of fascin expressing using FACS. ShRNA were designed and used especially for the activation experiments where cells need to be seed in serum containing media before stimulation in serum-free media. This could not be done using the SiRNA approach as they were generated in a serum-free media.

### Generation of eGFP-fascin expressing cell stable transfectants

Wild-type or mutant fascin-GFP fusion constructs were previously described [24] and kindly provided to us by Dr. Josephine C. Adam (Cleveland Clinic Foundation, Ohio, USA). Briefly, mutation in fascin was generated by substituting the major site that is phosphorylated by PKCα (Serine 39) with Aspartic Acid. After transfection using the Nucleaofector reagent (Amaxa BioSystems), stable clones were isolated post selection with G418 for 2–3 weeks followed by cloning. Fascin expression levels and mean florescent intensity (MFI) were always higher in GFP positive cells as assessed by FACS and western blots, respectively.

### Chemotaxis and invasion assay

The migration and metastatic potential of tumor cells were evaluated using the 24-well BD migration and BioCoat Matrigel Invasion Chamber, respectively, as per the manufacturer guideline (BD Bioscience). Cells (1–2×10^5^/well) were allowed to migrate for 18 hours and membranes were stained with a Diff Quick stain and mounted on slides. Cells that had migrated to the underside of the filter were counted using light microscope (Zeiss Axio Observer) in 5 randomly selected fields (magnification; 40x). Each assay was performed in triplicate and repeated at least 5 times. Due to variation in the number of migrated cells from different experiment, the results are normalized to control cells and the relative migration or invasion is expressed as mean ± SD of migrating cells relative to control cells.

### Purification of total RNA and RT-PCR

Total RNA was extracted from cells using RNAeasy Mini kit (QIAGEN). cDNA was synthesized using First Strand cDNA synthesis kit (Invitrogen). The relative mRNA copy number of a gene was determined by RT-PCR using molecular beacon method and detected by ABI PRISM 7900HT (Applied Biosystems) and expressed as previously described [25]. Sequences of forward, reverse and probe used in this experiment are shown in Table S1.

### Western Blot

Cells were washed with cold PBS, total proteins were extracted using RIPA buffer containing protease inhibitors, and concentrations were determined using Bradford Assay (Bio-Rad). Proteins (20 µg) were loaded onto an SDS-PAGE gel and transferred using semidry electrobloting (Bio-Rad). Membranes were then incubated in 5% (w/v) skimmed milk before incubation with primary antibodies overnight at 4°C. After washing, membranes were incubated with horseradish peroxidase-conjugated secondary antibody for 1 hour. Chemiluminescence Super Signal System (Thermo Scientific) was used for subsequent detection of bound antibodies.

For activation, exponentially growing cells were stimulated with 10 ng/ml of TNF-α in serum-free media for the indicated time points in the figures. Nuclear and cytoplasmic fractionation was done as previously described [26]. Proteins (15 µg of cytoplasmic and 10 µg of nuclear extract) were loaded and assayed as above. Equal amounts of supernatants were loaded onto an SDS-PAGE gel and protein expression was detected by western blot as described above. Gelatin zymography was done as previously described [27].

### Dual-luciferase reporter assay

NF-κB luciferase activity in cell lysates was measured using dual-luciferase reporter assay system (Promega) previously described [28]. pXPG-hIL-8p luciferase reporter plasmid was a generous gift from Dr. Abbas Hawwari, King Faisal Specialist Hospital and Research Centre, Riyadh, Saudi Arabia. Each experiment group was done in triplicate and repeated at least 5 times. *Firefly* luciferase was divided by the *Renilla* luciferase activity to normalize for transfection efficiency and the relative values are presented as fold change over non-stimulated control.

### Immunohistochemistry

Formalin-fixed, paraffin-embedded breast cancer sections of 71 patients deparaffinized and rehydrated. Antigen retrievals were done by microwaving for 15 minutes in a special citrate solution pH 6 (Dako). Endogenous peroxidase were blocked for 15 minutes with 3% hydrogen peroxide (Sigma) in methanol. Sections were then blocked with 10% goat serum (Sigma) for 60 minutes, followed by addition of a primary mouse anti-human fascin (1/200) or rabbit anti-BRMS1 (1/1000) antibodies for overnight incubation at 4°C. After washing, sections were incubated with labeled Polymer (EnVision^+^) HRP detection kit (Dako) for 30 minutes at room temperature. The HRP was detected using DAB substrate (Novocastra) for 4 minutes and the sections were counterstained for 1 minute with Instant hematoxylin (Shandon). The intensity of staining and the percentages of fascin and BRMS1 positive cells were quantified at 5 to 10 increments by an anatomical pathologist (AT) who had no prior knowledge of patient details. Type of breast cancer was confirmed at the time of reading. Histological grades of breast cancer sections were evaluated according to Scarff-Bloom-Richardson (SBR) classification [29].

For immunoflorescence staining of BRMS1, the above method of fixation and primary staining was used followed by Alexa-555 anti-rabbit secondary antibody. F-actin staining was done as previously described [30]. For cellular localization of BRMS1 or F-actin detection, 100 cells were assessed using attovision software on Pathway 855 (BD, Mountain View, CA).

### Statistical Analysis

The significance (0.05) of relationship between fascin expression and patient's clinicopathological parameters was assessed using Fisher exact test. The software package SAS 9.1 (SAS Institute, Cary, NC) was used for these analyses.

## Results

### Fascin expression in breast cancer is associated with poor prognosis, metastasis and reduced disease-free survival

**Figure 1:**
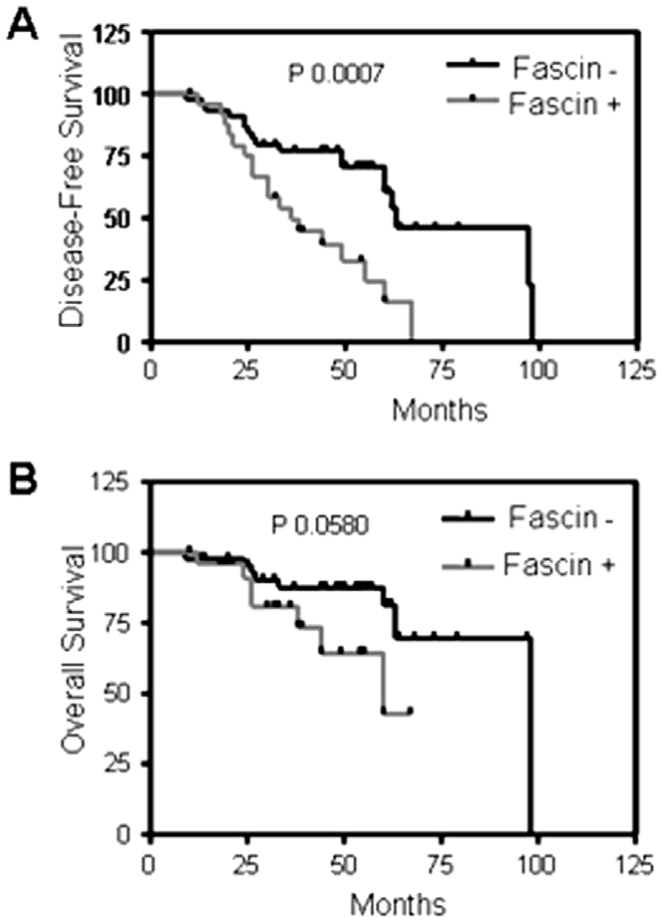
Fascin is associated with disease-free survival in human breast cancer samples. Survival with or without the disease was reported by oncologist and blotted in relation to fascin expression. Survival curves showing decreased disease-free (A) or overall (B) survival in patients that have fascin positive tumor.

Fascin expression in breast cancer correlates with poor prognosis of the disease and shorter disease-free and overall survival [21]. Here we have used immunohistochemistry to reexamine the relationship between the expression of fascin and metastasis in 71 breast cancer patients, which were diagnosed with invasive ductal carcinoma. Fascin was negative in normal breast luminal cells, but weak to moderate expression was seen in the myoepithelial and endothelial cells (data not shown), consistent with previous study [21]. In breast cancer samples however, fascin was expressed in the tumor cells of 40.84% of breast cancer patients. There were a strong correlation between fascin expression and; basal-like phenotype (<0.001), hormone receptor-negative (ER^−^, P<0.001), (PR^−^, P = 0.020), larger tumor size (P = 0.034), high histological grade tumors (P = 0.091), known poor prognostic markers (Table 1). Interestingly, fascin also significantly correlated with increased expression level of B7-H1 (P = 0.008), a T cell inhibitory molecule that is associated with bad prognostic makers in breast cancer [31]. Importantly, our data showed significant (P = 0.017) correlation between fascin expression and local as well as systemic metastasis (Table 1). In addition, there was a highly significant (P<0.001) association between fascin expression and decreased disease-free survival (Figure 1A), but the association with the overall survival (Figure 1B) was borderline significant (P = 0.058). Our in vivo findings demonstrate a strong correlation between fascin induction in breast cancer cells and; poor prognostic markers, increased tumor metastasis and reduced disease-free survival.

**Table 1:** ^
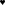
^Correlation between fascin expression in tumor cells and the clinico-pathological parameters of 71 breast cancer patients.

	Fascin		
	Negative	Positive	* *P*
**Age**			
<40 years	16 ^♣^(76)	5 (24)	0.575
≥40 years	33 (66)	17 (34)	
**Tumor Size**			
<4 CM	31 (79)	8 (21)	**0.035**
≥4 CM	18 (56)	14 (44)	
**Histological Grade (SBR)**			
I	1 (50)	1 (50)	0.091
II	27 (82)	6 (18)	
III	21 (58)	15 (42)	
**Her2/neu status**			
0	23 (68)	11 (32)	0.072
1	0 (0)	2 (100)	
2	10 (91)	8 (33)	
3	16 (67)	8 (33)	
**ER status**			
Negative	9 (39)	14 (61)	**<0.001**
Positive	70 (83)	5 (17)	
**PR status**			
Negative	20 (56)	16 (44)	**0.020**
Positive	29 (83)	6 (17)	
**B7H1**			
Negative	49 (72)	19 (28)	**0.008**
Positive	0 (0)	3 (100)	
**Basel**			
Negative	46 (77)	14 (23)	**<0.001**
Positive			
♦ **Local and systematic metastasis**			
Negative	25 (89)	3 (11)	**0.017**
Local	4 (80)	1 (20)	
Systemic	15 (56)	12 (27)	
**Neo-adjuvant Chemotherapy**			
Without	20 (67)	10 (33)	0.714
With	29 (69)	22 (31)	

**Abbreviations**:

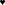
When interpreting data and correlating it with clinico-pathological parameters, the 5% expression of cells was the cut-off point below which were considered as negative and above as positive.

♣ Numbers between brackets are the percentages of patients,

♦ 11 patients were reported as no show or dead,

*P values in bold represent a significant data.

### Fascin regulates the morphology and migration of breast cancer cells

**Figure 2:**
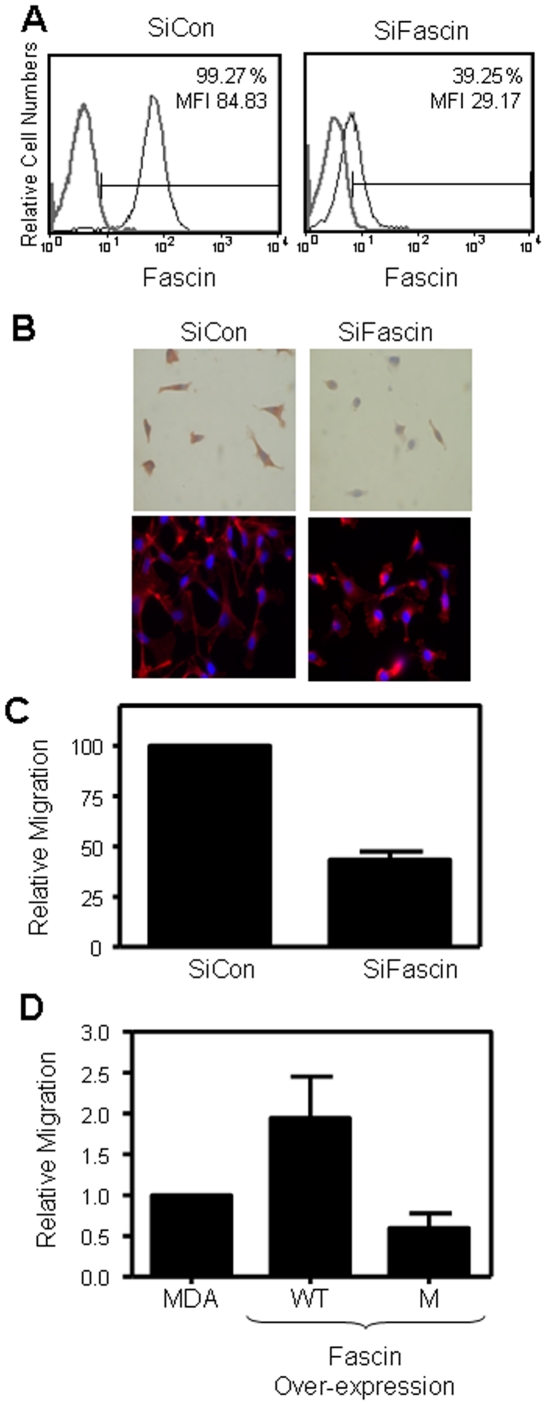
Fascin knockdown alters cell morphology and reduces cell migration. MDA-MB-231 cells were treated with control (SiCon) or Fascin (SiFascin) SiRNA for 72 hours. A) Cells were then collected and stained for fascin before detection by flow cytometry. B) Top: Immunohistochemistry showing cell morphology and fascin expression after treatment with SiCon or SiFascin in 8-well chamber. Bottom: Immunoflorescent staining showing the altered morphology and distribution of F-actin (red) in cells after treatment with SiFascin in 8-well chamber. Blue color indicates nuclear stain (Dabi). C) Bar graph showing reduced migration in fascin knockdown MDA-MB-231 cells. Data was normalized to SiCon cells and the relative migration is expressed as mean ± SD of triplicate experiments. D) Bar graph showing enhanced cell migration in MDA-MB-231 cells that over-express wild type (WT) fascin compared with mutant (M) over-expressing cells. Data was normalized to parental MDA-MB-231 and the relative migration is expressed as mean ± SD of triplicate experiments.

To address the relationship between fascin expression and metastasis we measured parameters important for this process using MDA-MB-231 breast cancer cell line as a model. This cell line is known to be ER/PR-negative, a feature of breast cancer cells with more invasive and motile phenotype [32]–[34]. FACS analysis (Figure 2A) and western blot (Figure S1A) demonstrate that these cells are fascin positive. To study fascin function in MDA-MB-231 cells, we transiently down-regulated its expression using fascin-specific siRNA (SiFascin). FACS histogram (Figure 1A) shows that 60–70% of the SiFascin treated cells became fascin-negative compared with the control-siRNA (SiCon) treated group. The mean florescence intensity (MFI) of SiFascin-treated cells was also reduced by greater than 60%. Similar to the FACS data, western blot indicated 70% inhibition of fascin in the knockdown cells (Figure S1A). Inhibition of the house keeping gene (GAPDH) using specific SiRNA was used as positive control in all of the assays (data not shown).

We examined whether fascin expression in human breast cancer cells induces morphological changes, as we have previously observed in mouse immune cells [22]. In culture, fascin-knockdown cells lost their typical/normal spindle shape that is seen in the control cells as shown by phase contrast inverted microscope (Figure S1B). Furthermore, immunohistochemistry staining demonstrated that while the control cells expressed fascin and have a spindle shape with fine dendrites, fascin-knockdown cells expressed lower fascin levels and exhibited more rounded morphology (Figure 2B Top). Immunoflorescent staining of SiFascin cells demonstrated these alterations in cell morphology and showed changes in the distribution of F-actin (Figure 2B Bottom). Fascin expression had no effect on MDA-MB-231 cell proliferation as assessed using the cell proliferation reagent WST1 and CSFE dye (data not shown).

We examined if fascin-mediated morphological changes affect breast cancer cell motility. Migration of the fascin-knockdown cells was significantly (P<0.001) inhibited by more than 50% compared with control cells (Figure 2C). Furthermore, SiFascin cells were also less migratory after 72 hours using the traditional wound healing assay (Figure S2). Difference in wound closure was not a reflection of cell division as similar number of cells was seeded and became confluent at the same time before the wound was made. To specifically link fascin expression to enhanced migration, we have generated MDA-MB-231 cells stably over-expressing wild-type (WT) or mutant fascin. Contrary to fascin-knockdown data, over-expression of WT (Figure S3) and not mutant fascin significantly (P = 0.01) enhanced MDA-MB-231 cell migration (Figure 2D). Collectively, our data shows that fascin expression in breast cancer cells regulates their morphology and migratory potential.

### Fascin regulates breast cancer cell invasion

**Figure 3:**
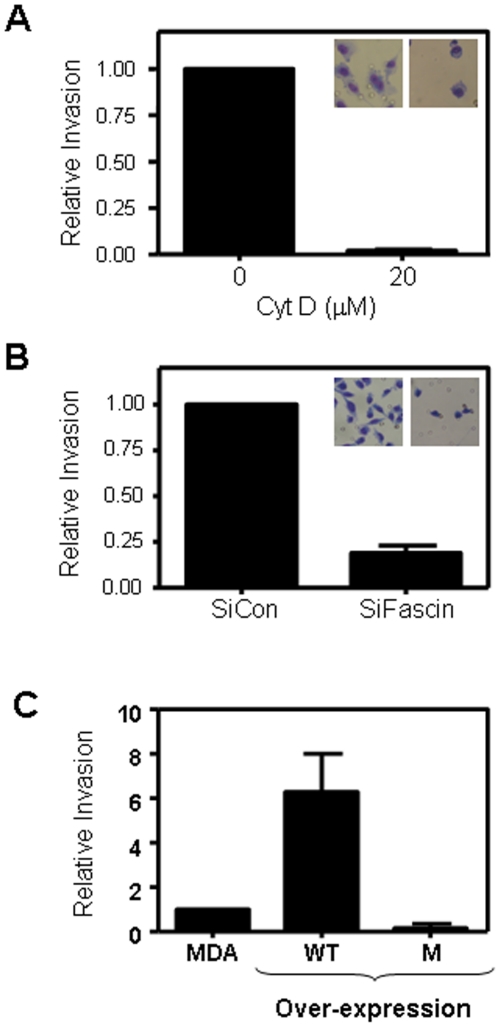
Fascin regulates breast cancer cell invasion. A) Bar graph showing inhibition of MDA-MB-231 cell invasion after pretreatment with 20 µM of Cytochalsin D (Cyt D) for 30 minutes prior to the assay. Data was normalized to untreated cells and the relative invasion is expressed as mean ± SD of triplicate experiments. B) Bar graph showing inhibition of MDA-MB-231 cell invasion after fascin knockdown with SiRNA. Data was normalized to con SiRNA cells and the relative invasion is expressed as mean ± SD of triplicate experiments. C) Bar graph showing enhanced cell invasion in MDA-MB-231 cells that over-express WT fascin. Data was normalized to parental MDA-MB-231 and the relative invasion is expressed as mean ± SD of triplicate experiments.

Fascin involvement in mediating breast cancer metastasis was then tested using a well-established invasion assay. Global actin polymerization inhibitor (Cytochalasin D), at a dose that did not effect cell viability (data not shown), altered MDA-MB-231 morphology and significantly (P<0.001) suppressed their invasion by greater than 95% (Figure 3A), consistent with the role of actin cytoskeleton in this process. Similarly, fascin-knockdown MDA-MB-231 showed significantly (P<0.001) impaired invasion and altered morphology compared with control cells (Figure 3B). On the contrary, over-expression of WT and not mutant fascin in MDA-MB-231 cells significantly (P = 0.003) enhanced their invasive capability (Figure 3C). Interference with fascin expression in MDA-MB-231 had no effect on cell proliferation or survival (data not shown). Collectively, these data demonstrate that fascin expression in breast cancer cells is strongly associated with enhanced cell motility and invasiveness.

### Fascin inhibits nuclear expression of BRMS1 in breast cancer cells

**Figure 4:**
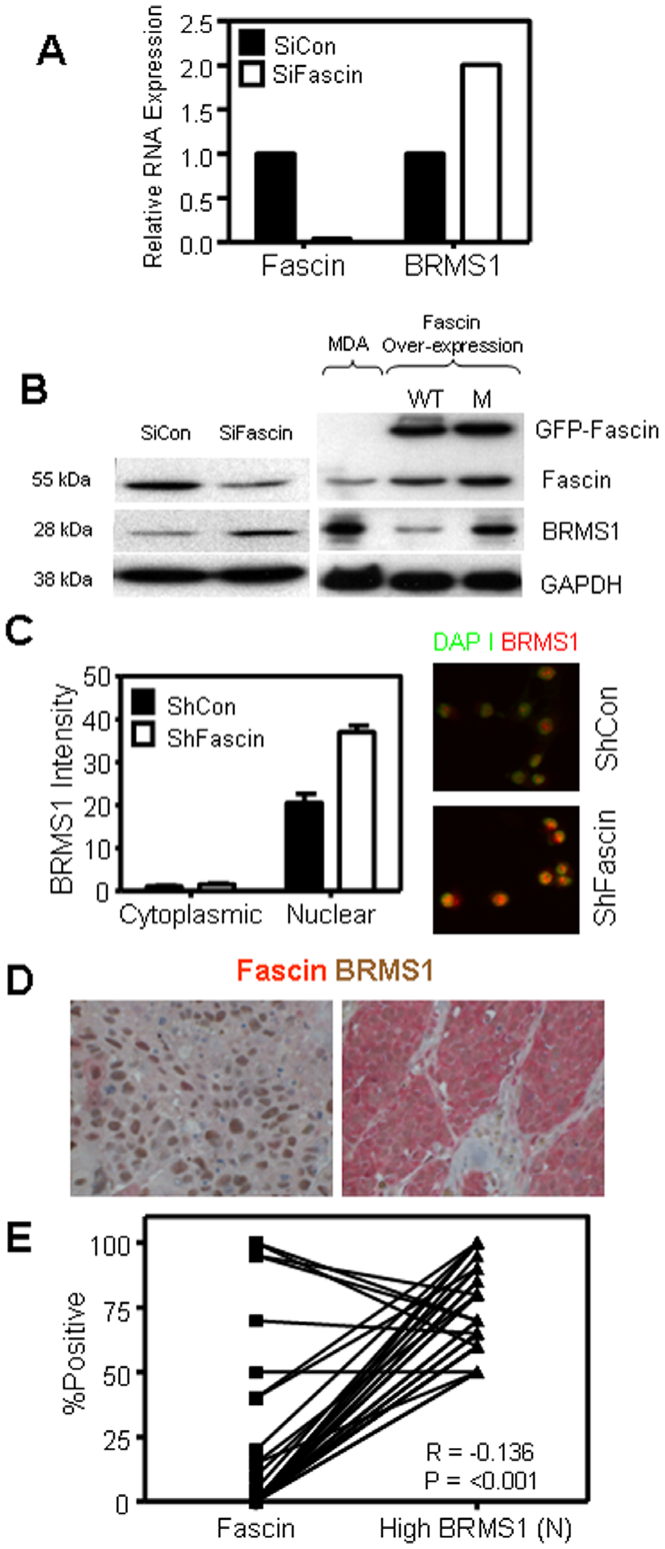
Fascin inhibits nuclear expression of BRMS1 in breast cancer cells. A) Bar graph showing the relative RNA expression as assessed by real time PCR. Fascin RNA was inhibited, while BRMS1 RNA was enhanced after fascin knockdown. B) Western blot showing enhanced BRMS1 protein expression when fascin was knockdown (Left) and BRMS1 inhibition when WT fascin was over-expressed (Right). C) Right; Representative photograph showing induction of BRMS1 expression in the nucleus after fascin knockdown. Left; Bar graph showing the mean intensity of BRMS1 expression in the nucleus after fascin knockdown as assessed on more than 100 cells per group using attovision software on Pathway 855 from BD. D) Paraffin-embedded sections from breast cancer patients were stained for fascin and BRMS1 and the expression profile and staining intensity were assessed by pathologist. Left: representative image showing strong BRMS1 nuclear staining (Brown) and weakfascin cytoplasmic staining (red). Right: representative image showing weak BRMS1 nuclear staining (Brown) and strong fascin cytoplasmic staining (red). E) Bar graph showing reduced levels of fascin in patients that express high level (≥50 positive with +3 intensity) nuclear BRMS1.

BRMS1 is a known metastasis suppressor in many cancer types including breast cancer [35]. We examined whether fascin-mediated breast cancer cell invasion has an effect on the expression levels and cellular localization of BRMS1. SiFascin breast cancer cells showed significantly enhanced BRMS1 RNA (Figure 4A) and protein (Figure 4B left) expression compared with SiCon, consistent with the role of fascin in enhancing breast cancer metastatic potential. Conversely, over-expression of WT fascin in MDA-MB-231 dramatically suppresses BRMS1 expression (Figure 4B right), further confirming the inverse relationship between these two proteins. BRMS1 expression was found to be predominantly in the nucleus and fascin-knockdown clearly leaded to enhanced expression of nuclear BRMS1 (Figure 4C). Examining the relationship between BRMS1 and fascin in our breast cancer patient samples demonstrated variations in the intensity and subcellular distribution of BRMS1 (Figure 4D). Most importantly, 36 patients that were scored as having high levels of nuclear BRMS1 (≥50% of tumor cells express high levels (intensity of +3) of nuclear BRMS1) showed reduced levels of fascin (Figure 4E), demonstrating an inverse relationship between fascin and nuclear BRMS1 that is statistically significant (P<0.001). This data demonstrates that fascin can directly or indirectly regulate the tumor suppressor BRMS1 nuclear expression.

### Fascin enhances metastasis-associated genes

**Figure 5:**
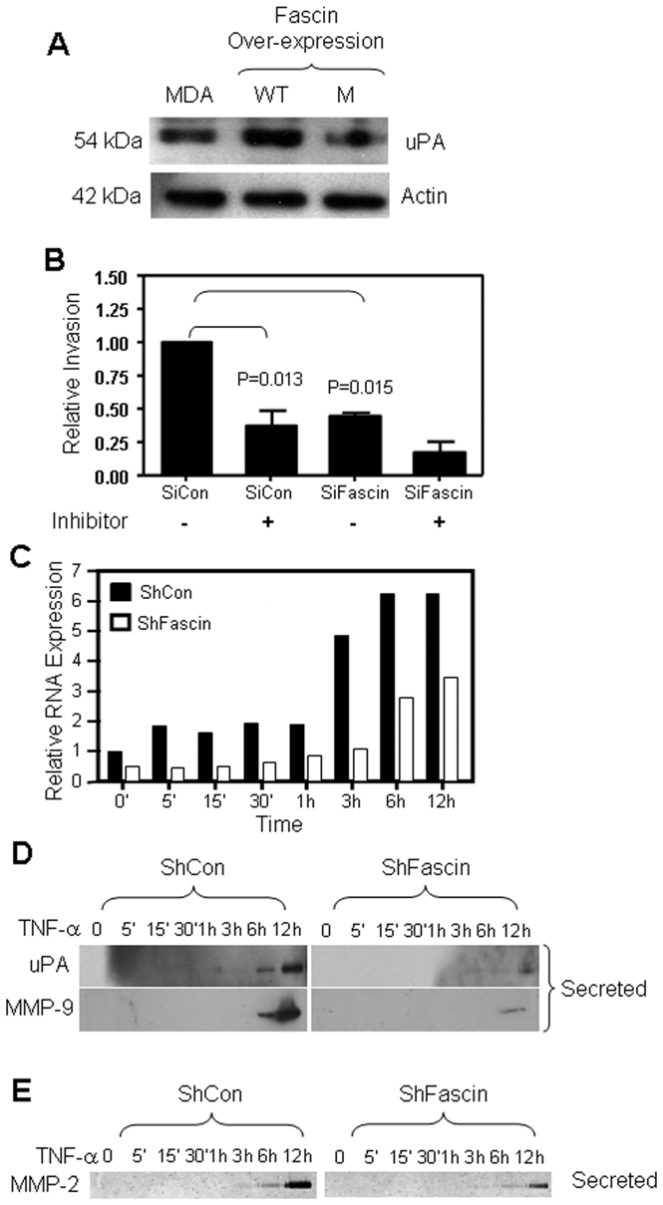
Fascin enhanced uPA, MMP-2 and MMP-9 expression. A) Western blot showing increased expression of uPA in MDA-MB-231 cells over-expressing WT fascin compared with the mutant over-expressing cells. B) Bar graph showing reduced invasion of SiCon-treated cells when MMP-2/MMP-9 inhibitor II (20 µM) was used. Data was normalized to untreated SiCon cells and the relative invasion is expressed as mean ± SD of triplicate experiments. C) Bar graph showing reduced RNA expression of uPA in fascin knockdown cells as assessed by real time PCR. D) Western blot showing reduced uPA (top) or MMP-9 (bottom) secretion in supernatants of fascin-knockdown cells. E) MMP-2 gelatinolytic activity showing reduced enzymatic activity of secreted MMP-2 in the supernatants of fascin-knockdown cells.

We tested whether fascin enhances metastasis via counteracting BRMS1 effect on key downstream mediators that are involved in this process such as *uPA* [12]. Consistent with enhanced invasion, cells that over-expressed WT and not mutant fascin showed higher levels of *uPA* expression (Figure 5A). To directly link invasion to well established mediators of metastasis, we blocked activity of MMP-2 and MMP-9, which are activated by *uPA* [9] and are among the most well established proteases known to degrade ECM and facilitate invasion and metastasis [36]–[38]. MMP-2 and −9 selective inhibitors significantly (P = 0.013) reduced the invasion of control cells to comparable level with the fascin-knockdown cells (Figure 5B), implicating a role for these proteases in fascin-mediated invasion. Cytochalasin D, which significantly diminished the cell invasion capability (Figure 3A), also inhibited the expression of MMP-2 and MMP-9 (Figure S4).

Since TNF-α was shown to trigger uPA expression in MDA-MB-231 cells [12], we activate our cells with TNF-α to further test the link between fascin and uPA secretion in the supernatants. Maximum induction of uPA RNA was seen at 6 hours post TNF-α stimulation in fascin-positive breast cancer cells, while fascin-knockdown cells failed to induce significant up-regulation compared with the control cells (Figure 5C). uPA protein secretion in control cells was detected in the supernatant 6 hours post TNF-α stimulation and notable reduction was observed in fascin-knockdown cells 12 hours post activation (Figure 5D). In parallel, MMP9 was also detected in control cells 6 hours post TNF-α stimulation and only detected at reduced levels 12 hours post TNF-α stimulation of fascin-knock down cells. Furthermore, reduced MMP-2 protein expression in fascin-knockdown cells that was observed above is in line with decreased TNF-α-mediated enzymatic activity in gelatin zymograms (Figure 5E).

### Fascin enhanced NF-κB activity

**Figure 6:**
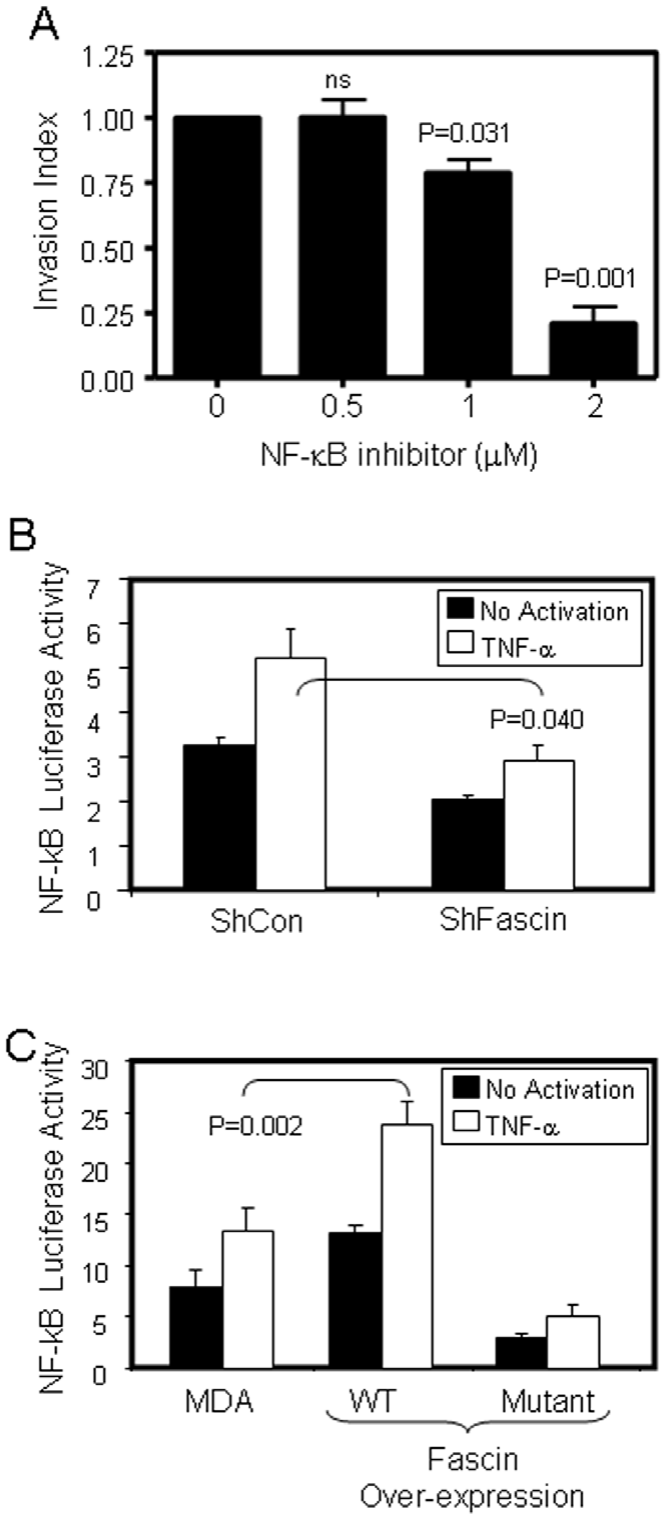
Fascin enhanced NF-κ -dependent transcriptional activity. A) Bar graph showing inhibition of MDA-MB-231 cell invasion that were treated with NF-κB inhibitor. Data was normalized to untreated cells and the relative invasion is expressed as mean ± SD of triplicate experiments. B) ShCon or ShFascin MDA-MB-231 cells were co-transfected with the NF-κB luciferase promoter and Renilla promoter as in methods. Cells were stimulated with or without (20 ng/ml) TNF-α and luciferase activity was assessed as in methods. C) MDA-MB-231 parental or cells that over-express WT or mutant fascin were co-transfected with the NF-κB luciferase promoter and Renilla promoter as in methods. Cells were stimulated with or without (20 ng/ml) TNF-α and luciferase activity was assessed as in methods.

Since uPA promoter was reported to contain an NF-κB binding site [39], we asked if fascin enhanced *uPA* secretion after TNF-α activation is mediated via enhancement of NF-κB activity. Inhibition of the NF-κB pathway significantly reduced cell invasion in a dose-dependent manner (Figure 6A), implicating the importance of NF-κB in this process. Importantly, fascin-knockdown cells showed significantly (P = 0.040) impaired NF-κB luciferase activity upon TNF-α activation when compared with control cells (Figure 6B). In contrast, cells that over-expressed WT fascin exhibited significantly (P = 0.002) enhanced NF-κB luciferase activity upon TNF-α activation (Figure 6C), indicating that fascin positively regulates NF-κB transcriptional activity. No NF-κB transcriptional activities was observed upon TNF-α activation, when mutant NF-κB-luciferase reporter was used (data not shown). Collectively, our data strongly demonstrated that fascin suppressed BRMS1 and enhanced NF-κB activity.

**Figure 7:**
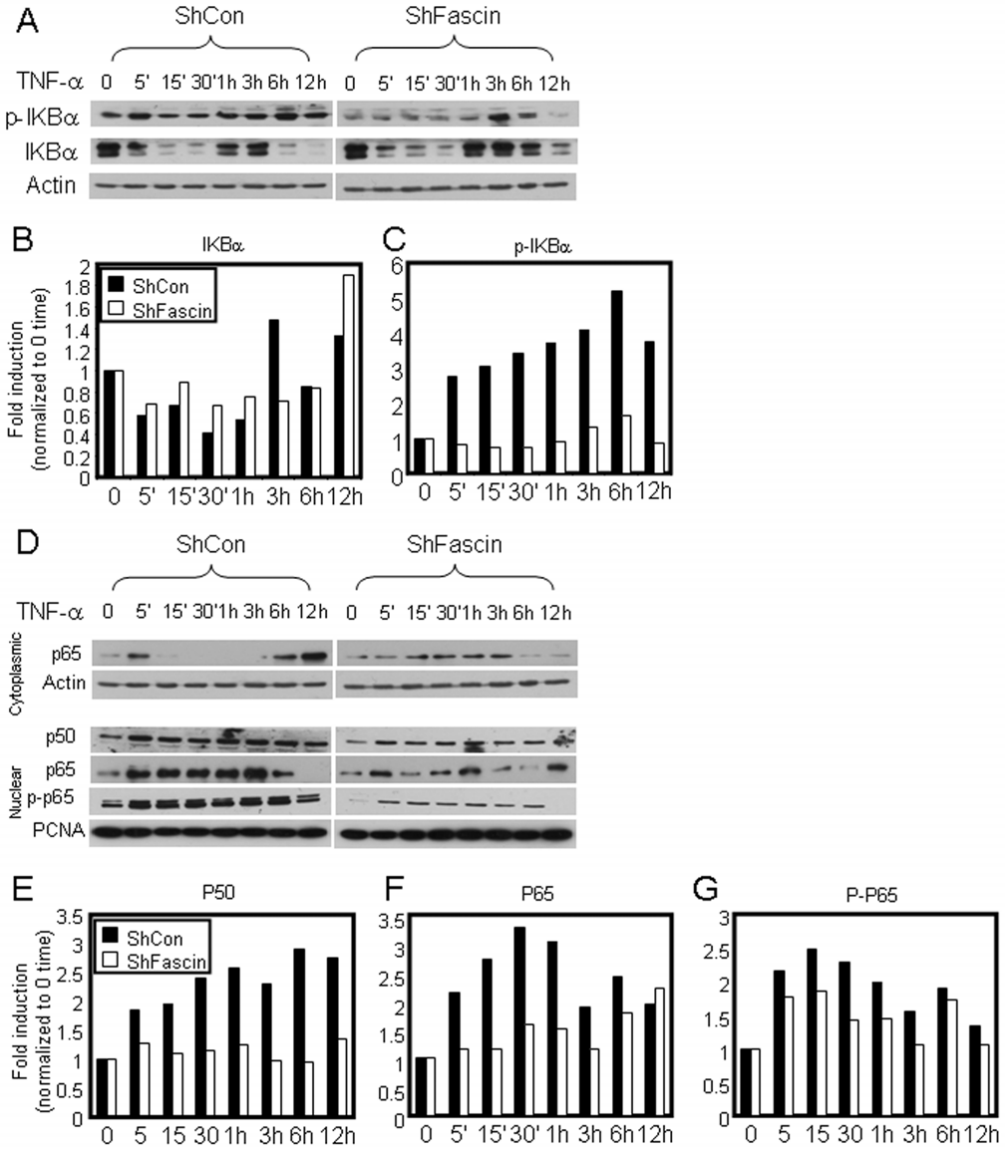
Fascin enhanced NF-κB nuclear translocation in MDA-MB-231. A) Western blot showing reduced phosphorylation of IKBα in response to TNF-α stimulation in fascin knockdown cells. B and C) Bar graph showing quantitation of total and phosphorylated IKBα in ShCon and ShFascin cells after stimulation with TNF-α for the indicated time. Results showed the mean of triplicate experiments after normalization to actin and each time point is normalized to 0 time. D) Western blot showing reduced nuclear translocation and phosphorylation of p65 in response to TNF-α stimulation in Fascin knockdown cells. E-G) Bar graph showing quantitation of nuclear P50 and P65 and phosphorylated P65 in ShCon and ShFascin cells after stimulation with TNF-α for the indicated time. Results showed the mean of triplicate experiments after normalization to PCNA and each time point is normalized to 0 time.

**Figure 8:**
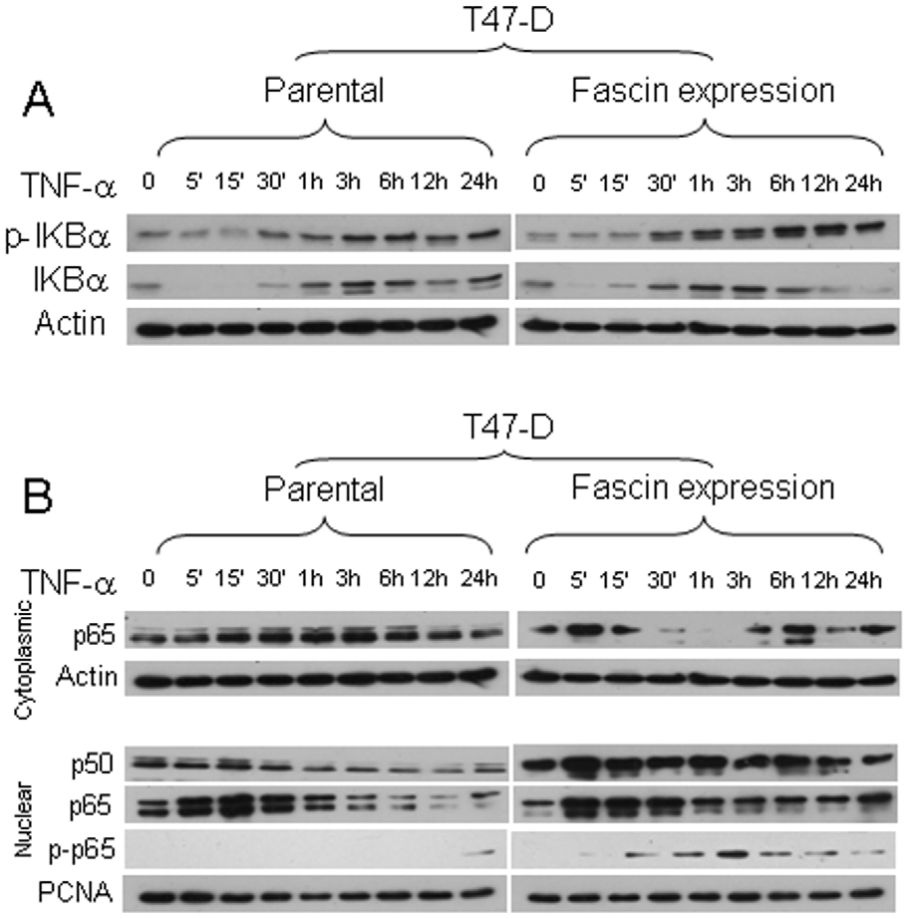
Fascin enhanced NF-κB nuclear translocation in T47-D. A) Western blot showing enhanced phosphorylation of IKBα in response to TNF-α stimulation in fascin-expressing T47-D cells. B) Western blot showing enhanced nuclear translocation and phosphorylation of p65 in response to TNF-α stimulation in fascin-expressing T47-D cells.

Phosphorylation of the inhibitory subunit (IκBα) leads to libration and translocation of NF-κB to the nucleus [40], [41]. We thus examine whether the observed fascin mediated NF-κB transcriptional activity is related to enhanced NF-κB nuclear translocation. The cytoplasmic levels of total IκBα were reduced in control and fascin knockdown cells in response to activation (Figure 7A). However, the magnitude of IκBα inhibition was more pronounce in the control cells especially at earlier time points (Figure 7B). There were a time dependent increase in IκBα phosphorylation in control cells after activation, which was severely reduced in the fascin-knockdown cells (Figure 7 A and C). The effect of fascin on IκBα phosphorylation and degradation have also been observed in another breast cancer cell line (T47-D). Expression of wild type fascin in T47-D cells, which are fascin negative (Figure S5A), reduced BRMS1 expression (Figure S5B). Interestingly, fascin expressing T47-D cells demonstrated reduced total levels of IκBα and enhanced magnitude of IκBα phosphorylation in response to activation especially at later time points (Figure 8A).

To test whether reduced IκBα phosphorylation in fascin knockdown cells would affect the phosphorylation of p65 and translocation of p65/p50 to the nucleus, cytoplasmic and nuclear fractions were extracted from cells after activation and the level of these proteins and the status of phosphorylation were evaluated. TNF-α activation induced time-dependent p65/p50 translocation into the nucleus in control and fascin knockdown cells (Figure 7 D, E and F). However, the levels of p65/p50 translocation into the nucleus in fascin knockdown cells were notably lower especially at the earlier time points, indicating less sustainable p65 nuclear translocation in fascin knockdown cells. There were also time-dependent increases of p65 phosphorylation in control and fascin knockdown cells. However, the magnitude of induction of p65 phosphorylation in fascin knockdown cells was notably lowered (Figure 7 D and G). In T47-D cells, TNF-α activation triggered early p65 translocation into the nucleus which declined after 30 minutes (Figure 8B). Nuclear translocation of p65 in response to TNF-α activation was more pronounced in the fascin expressing T47-D cells. Most importantly, the nuclear translocation of p65 after 30 minutes was more sustainable in fascin expressing T47-D cells. Furthermore, there were also time-dependent increases of p65 phosphorylation in fascin expressing T47-D cells, which was not seen in T47-D parental cells till 24 hours. Collectively, our data demonstrated that fascin positively regulate NF-κB nuclear translocation and transcriptional activity.

## Discussion

Metastasis and not the primary tumors remained the main causes of cancer mortalities [1], [2], stressing the need to understand the cellular and molecular mechanisms that regulate this process. It is a complex process where cytoskeletal proteins were reported to regulate multiple cellular processes including morphological changes and motility, which are critical steps for metastasis (Reviewed in [42]). In this study, we have shown an association between the actin-bundling protein (fascin) and expression of bad prognosis, metastasis and reduced disease-free survival. Our in vitro data demonstrated fascin involvement in regulation of breast cancer cell invasiveness and identifies some of the underlying molecular mechanisms.

Breast cancers mainly arise from the luminal compartment of the breast [43], but a small minority of tumors arise from multi-potent progenitor cells, which can differentiate into luminal or myoepithelial lineages [44], [45]. The aggressive breast cancer with a basal-like phenotype is triple negative (ER, PR and HER2) and express both luminal and myoepithelial markers [46]. Our breast cancer patients showed an association between fascin expression and basal-like phenotype and high histological grade tumors, a type of breast cancers that are associated with metastasis [46], [47]. Furthermore, our patients showed an association between fascin expression, increase metastasis and shorter disease-free survival and reduced nuclear BRMS1, consistent with the in vitro data. High fascin expression in our patients significantly correlated with expression of other poor prognostic markers of breast cancer such as tumor size and B7-H1. Whether fascin has a direct effect in regulating the poor prognostic markers of breast cancer remains to be elucidated.

NF-κB pathway is constitutively activated in many cancers, which leads to enhancement of metastasis (reviewed in[48]) and positively regulates the expression of *uPA* [39], which in turn can activate the MMPs to facilitate invasion by degrading the ECM [13]. BRMS1 negatively regulates *uPA* expression through inhibition of the NF-κB activity in breast cancer and melanoma cells [12]. Consistent with those findings, our data showed that fascin expression in breast cancer cells, which enhances invasion, suppresses BRMS1 and counteract its effect on downstream targets by increasing NF-κB activity, *uPA* secretion and MMP enzymatic activity. Although we could not conclude whether fascin suppression of BRMS1 is directly responsible for the inhibition of BRMS1 downstream targets, we demonstrated that fascin regulates MMPs expression and activation, key molecules that facilitate invasion and metastasis. Furthermore, TNF-α stimulation was shown to induce over-expression of fascin that in turn up-regulate MMP-9 expression in cholangiocarcinoma [49].

While our data demonstrated a suppression of BRMS1 by fascin, the exact mechanism by which this mediated has not been elucidated. Interestingly, previous study reported down-regulation of fascin when they expressed BRMS1 in a human ovarian carcinoma, but the supporting data was not presented [50]. Both Zhang *et al* and our findings support the existence of a direct or indirect interaction between fascin and BRMS1. Fascin is mainly a cytoplasmic protein with higher expression at the submemebrane and around the nuclear membrane as shown in our work and reviewed by Kureishy N *et al* [51]. Although BRMS1 is predominantly nuclear, it is expressed in the cytoplasm as shown by our data and by Frolova N *et al* [52]. It is therefore possible that there is an active shuttling of this molecule between the cytoplasm and the nucleus, where its translocation is controlled by direct or indirect interaction with fascin molecules.

Our study showed that fascin acts at several cellular fronts to facilitate cell invasion. It may act by negatively regulating the expression of BRMS1, thereby enhancing NF-κB activity and subsequent augmentation of *uPA* and MMPs expression. It is also possible that fascin and BRMS1 have a feedback loop where BRMS1 may also down-regulate fascin to inhibit cancer cells from metastasis. This assumption is supported by a study where BRMS1 was demonstrated to down-regulate fascin expression in human ovarian carcinoma cell line without showing data [50], and concluded that this could be a potential mechanism underlying BRMS1 suppression of metastasis. Alternatively, fascin may act via yet unknown pathway to positively regulate NF-κB transcriptional activity, which in turn enhances the expression of *uPA* and MMPs.

This study demonstrates a clear role for fascin in regulating breast cancer invasion partially through modifying the expression of metastasis-associated genes, making fascin a good target for therapeutic intervention in metastatic breast cancer cells. The relevance of our finding becomes more significant after the recent finding of a synthetic compound (migrastatin) that specifically targets fascin and inhibits its metastasis-mediated activity [53]. Identifying the specific molecular mechanism of fascin regulation of breast cancer invasion and metastasis will have a great impact on designing a better therapy for breast cancer metastasis.

## Supporting Information

Figure S1 **A) Insert is the western blot showing fascin (top) or GAPDH (bottom) in MDA-MB-231 cells after treatment with SiCon and SiFascin and bar graph showing the level of expression of fascin after normalization to GAPDH as assessed by densitometry.** B) Morphology of MDA-MB-231 cells in culture after treatment with SiCon and SiFascin.
(TIF)

Figure S2 **A) Wound healing assay showing wound healing impairment in SiFascin treated cells compared with the SiCon cells.** B) Bar graph showing measurements of the wound size in ShCon and ShFascin MDA-MB-231 cells at the indicated time points. Measurements were collected from 3 different sites and normalized to the size at 0 time.
(TIF)

Figure S3 **Flow cytometry histogram showing increase MFI of fascin in MDA-MB-231 over-expressing wild type (WT; gray line) compared with the parental cells (MDA-MB-231; black line).**
(TIF)

Figure S4 **Western blot showing reduced expression levels of MMP-2 and MMP-9 in MDA-MB-231 cells after treatment with Cytochalasin D (20 µM) for 30 minutes.** Cells were washed and cultured for over night prior to harvesting for western blot.
(TIF)

Figure S5 **A) FACS dot plot showing fascin expression in T47-D cells after transfection with wild type GFP-fascin.** Cells were then stained with APC-labeled anti-fascin antibody to confirm fascin expression in GFP-positive cells. B) Western blot showing reduced expression of BRMS1 in fascin expressing T47-D cells.
(TIF)

Table S1 **Table showing the sequences of forward and reverse primers as well as the probes of the different genes that were used in this experiment.**
(DOC)
