# Supplementary figures and images for: Fascin Is a Key Regulator of Breast Cancer Invasion That Acts via the Modification of Metastasis-Associated Molecules
Source: PLoS One. 2011 Nov 4;6(11):e27339. doi: 10.1371/journal.pone.0027339 (PMC3208623; doi:10.1371/journal.pone.0027339)

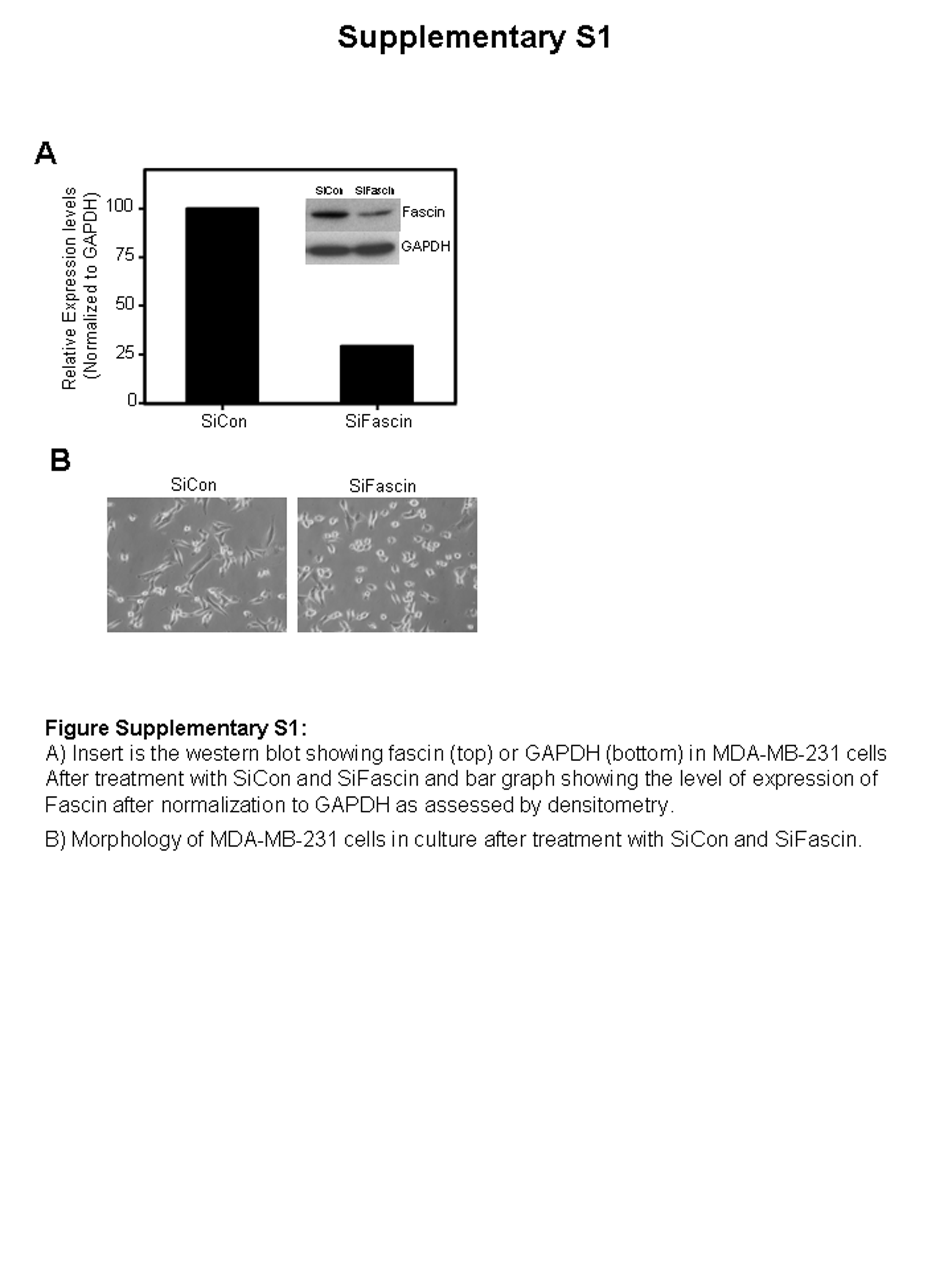

Supplement: Figure S1 — A) Insert is the western blot showing fascin (top) or GAPDH (bottom) in MDA-MB-231 cells after treatment with SiCon and SiFascin and bar graph showing the level of expression of fascin after normalization to GAPDH as assessed by densitometry. B) Morphology of MDA-MB-231 cells in culture after treatment with SiCon and SiFascin. (TIF) [file pone.0027339.s001.tif]

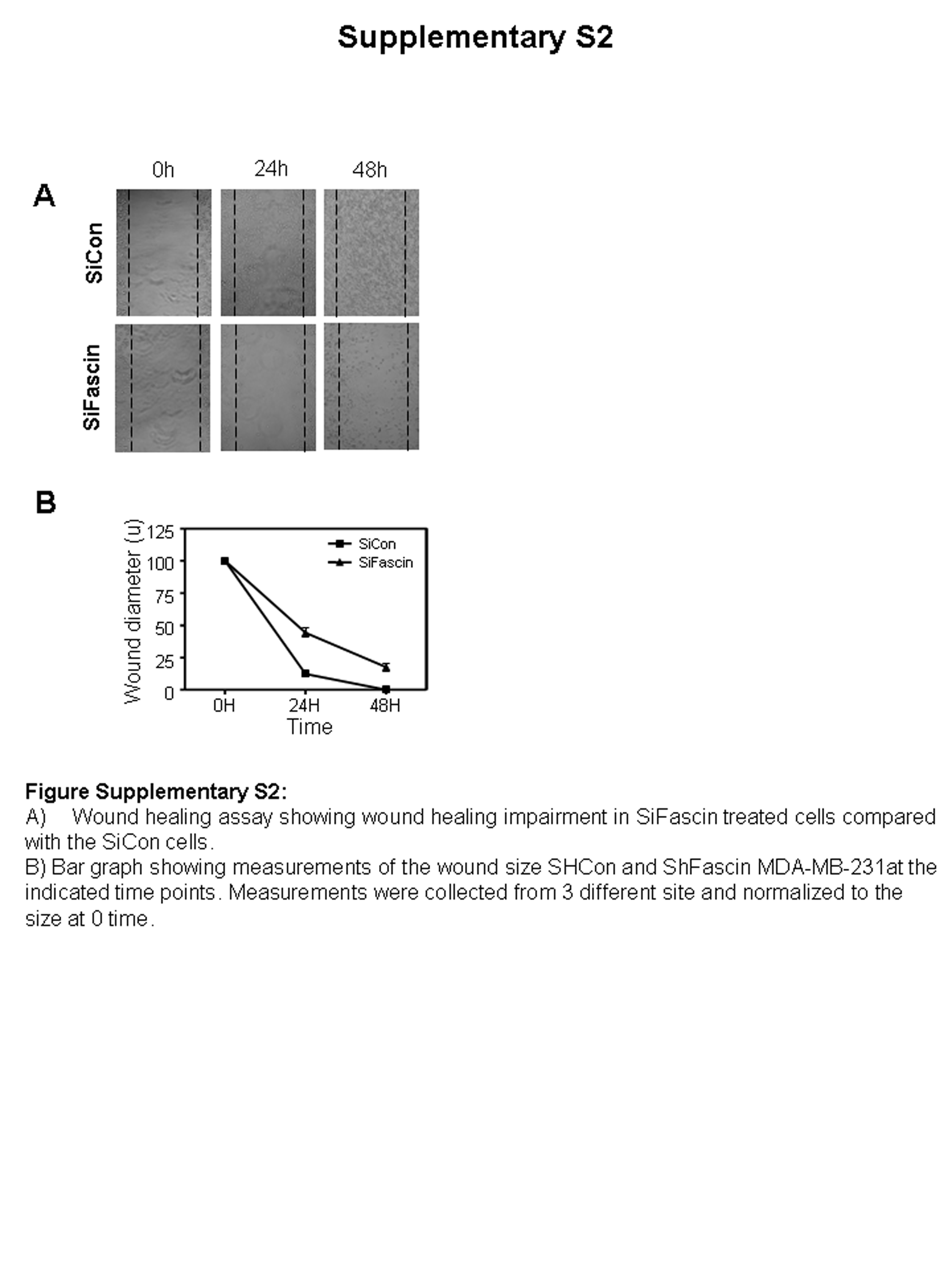

Supplement: Figure S2 — A) Wound healing assay showing wound healing impairment in SiFascin treated cells compared with the SiCon cells. B) Bar graph showing measurements of the wound size in ShCon and ShFascin MDA-MB-231 cells at the indicated time points. Measurements were collected from 3 different sites and normalized to the size at 0 time. (TIF) [file pone.0027339.s002.tif]

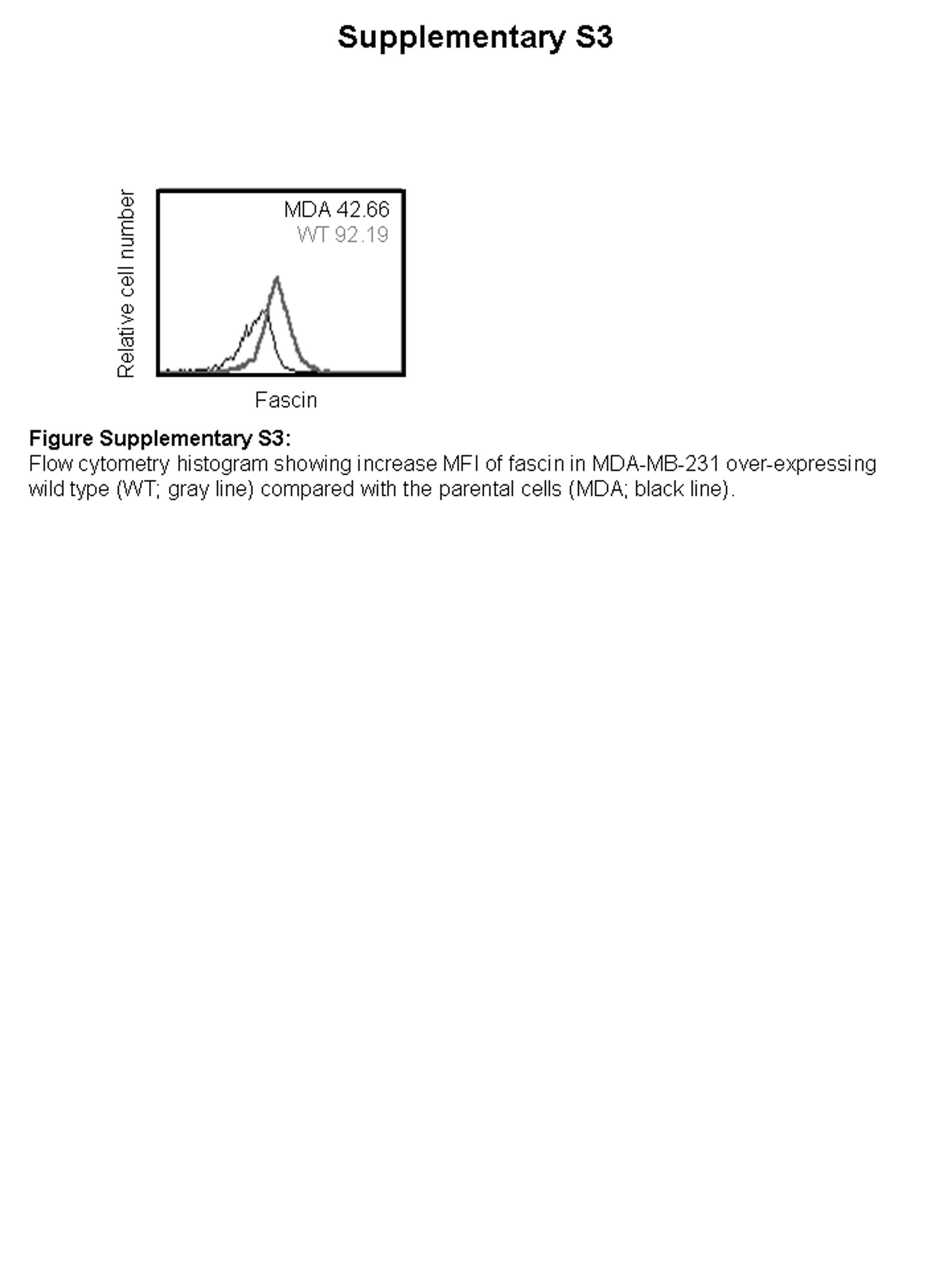

Supplement: Figure S3 — Flow cytometry histogram showing increase MFI of fascin in MDA-MB-231 over-expressing wild type (WT; gray line) compared with the parental cells (MDA-MB-231; black line). (TIF) [file pone.0027339.s003.tif]

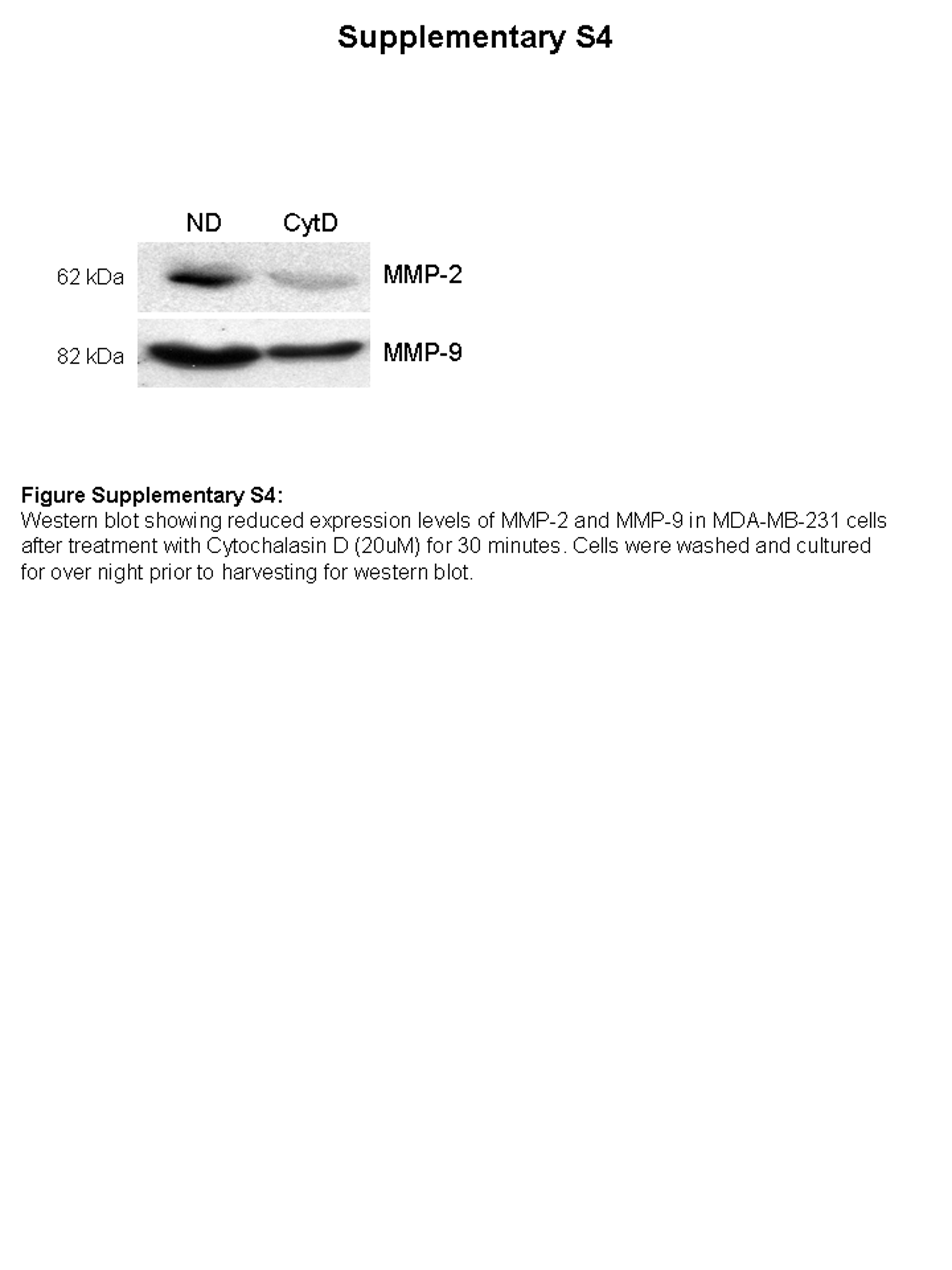

Supplement: Figure S4 — Western blot showing reduced expression levels of MMP-2 and MMP-9 in MDA-MB-231 cells after treatment with Cytochalasin D (20 µM) for 30 minutes. Cells were washed and cultured for over night prior to harvesting for western blot. (TIF) [file pone.0027339.s004.tif]

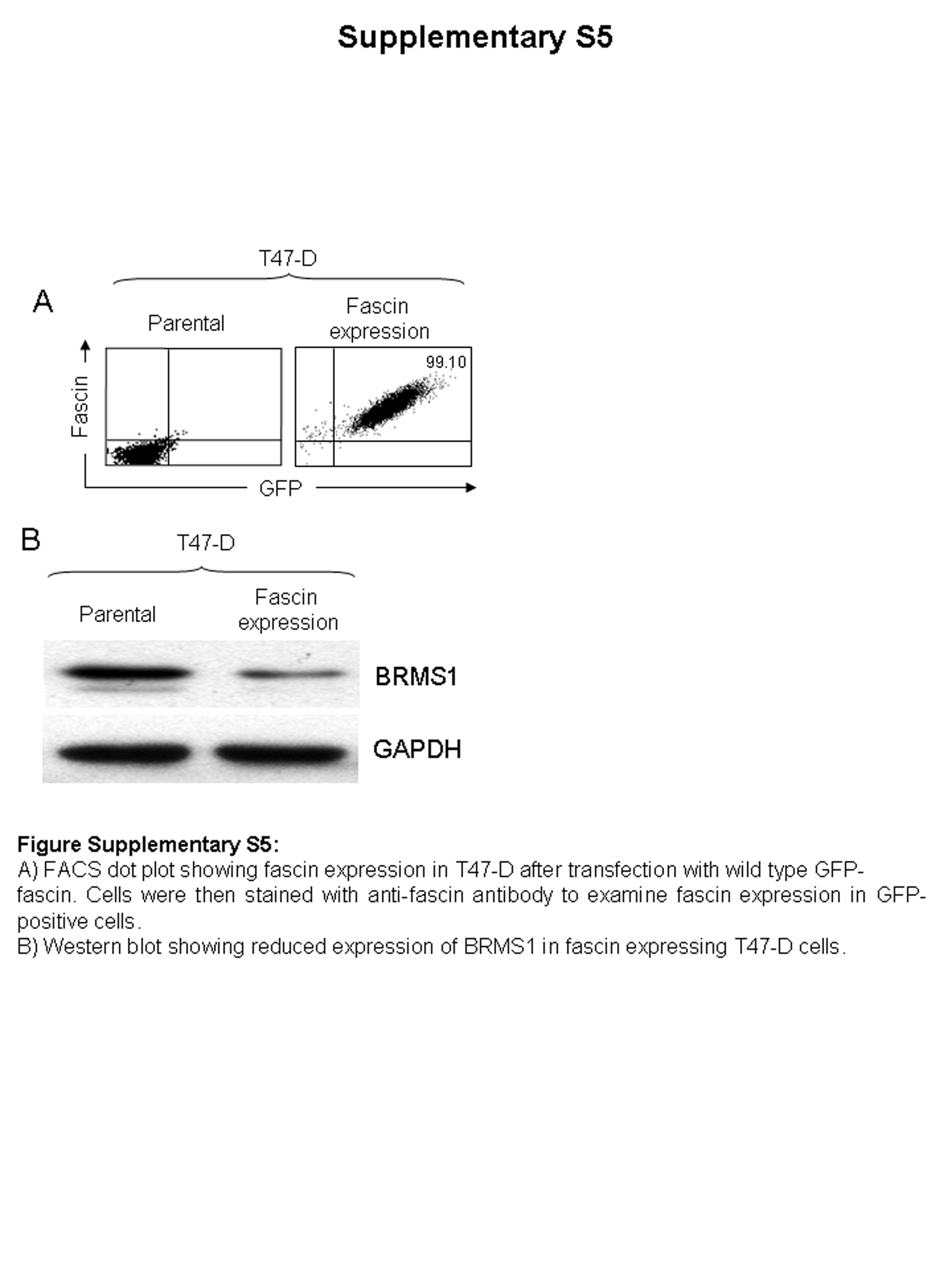

Supplement: Figure S5 — A) FACS dot plot showing fascin expression in T47-D cells after transfection with wild type GFP-fascin. Cells were then stained with APC-labeled anti-fascin antibody to confirm fascin expression in GFP-positive cells. B) Western blot showing reduced expression of BRMS1 in fascin expressing T47-D cells. (TIF) [file pone.0027339.s005.tif]
