# Supplementary material for: Fascin Is a Key Regulator of Breast Cancer Invasion That Acts via the Modification of Metastasis-Associated Molecules
Source: PLoS One. 2011 Nov 4;6(11):e27339. doi: 10.1371/journal.pone.0027339 (PMC3208623; doi:10.1371/journal.pone.0027339)
Supplement: Table S1 — Table showing the sequences of forward and reverse primers as well as the probes of the different genes that were used in this experiment. (DOC) [file pone.0027339.s006.doc]

**Table S1**

| **Gene** | **Forward** | **Reverse** | **Probe** |
| --- | --- | --- | --- |
| **Beta-actin** | CACCACACCTTCTACAATGA | AGGTCTCAAACATGATCTGG | Fam-CCGCTCGCGAGAAGATGACCCAGATCATGTTTGAGACCTGAGCGG-Dabcyl |
| **Fascin** | CCAGCTATGACGTCTTCCAG | TCGAAGAAGAAGTCCACAGG | Fam-CCGCTCCGCCTACAACATCAAAGACTCCACAGAGCGG-Dabcyl |
| **BRMS1** | AATGGTGGGATGACAAACT | GAAGCATGTCACGATGTA | Fam-CCGCTCCCTCTGGTTTCTGGCCCATACATCGTGACAGAGCGG-Dabcyl |
| **uPA** | CCTCATCCTACACAAGGA | ACTGGGGATCGTTATACA | Fam- GCCGGACATTGCCTTGCTGAAGATCCGCCGGC-Dabcyl |
